# Routine Immediate Postoperative Chest Radiography After Minimally Invasive Pulmonary Lobectomy Has a Low Impact on Clinical Management: A Value-Based Imaging Analysis

**DOI:** 10.3390/tomography12090136

**Published:** 2026-09-17

**Authors:** Nicola Rotolo, Andrea Imperatori, Alberto Colombo, Benedetta Bazzoli, Luca Filipponi, Filippo Piacentino, Federico Fontana

**Affiliations:** 1 Research Center for Minimally Invasive Surgery, Department of Medicine and Technological Innovation, University Hospital of Insubria—Asst-Settelaghi, 21100 Varese, Italy; bbazzoli@studenti.uninsubria.it; 2 Research Center for Thoracic Surgery, Department of Medicine and Surgery, University Hospital of Insubria—Asst-Settelaghi, 21100 Varese, Italy; andrea.imperatori@uninsubria.it (A.I.); acolombo1@studenti.uninsubria.it (A.C.); 3 Thoracic Surgery Unit, University Hospital of Insubria—Asst-Settelaghi, 21100 Varese, Italy; luca.filipponi@asst-settelaghi.it; 4 Diagnostic and Interventional Radiology Unit, University Hospital of Insubria—Asst-Settelaghi, 21100 Varese, Italy; filippo.piacentino@asst-settelaghi.it (F.P.); federico.fontana@uninsubria.it (F.F.)

**Keywords:** chest X-ray, thoracic surgery, video-assisted thoracoscopic surgery (VATS), pulmonary lobectomy, postoperative care, Enhanced Recovery After Surgery (ERAS), diagnostic yield

## Abstract

Background/Objectives: Despite the widespread adoption of minimally invasive video-assisted thoracoscopic surgery (VATS) for pulmonary lobectomy and Enhanced Recovery After Surgery (ERAS) protocols, routine chest X-rays (CXRs) in the Post-Anesthesia Care Unit (PACU) after VATS pulmonary lobectomy remain common. Evidence questioning their clinical utility in asymptomatic patients is growing. This study aimed to evaluate the therapeutic yield of routine PACU CXRs following VATS lobectomy, in a homogeneous cohort (PACU time point). Methods: This single-center retrospective cohort study included 554 consecutive patients who underwent elective VATS lobectomy (2014–2025). The primary endpoint was the therapeutic yield of routine PACU CXRs, defined as the proportion directly leading to a documented change in postoperative management. Secondary outcomes included the yield of post-drain-removal CXRs, radiographic findings, and the yield in patients with intraoperative complications. Results: Among 554 routine PACU CXRs, only 12 (2.2%; 95% CI: 1.1–3.7) led to a management change; surgical re-intervention was required in 1 patient and chest tube reinsertion in 5 patients. By contrast, 93 post-drain-removal CXRs (16.8%; 95% CI: 13.7–20.0) led to a change (*p* < 0.001). The most frequent abnormal findings were pneumothorax (5.8%), atelectasis (4.5%), and pleural effusion (2.0%). Routine PACU imaging generated an estimated cost of €16.620 and a cumulative radiation exposure of 27.7 mSv. Conclusions: Routine PACU CXR after VATS lobectomy has a very low therapeutic yield and rarely changes management in asymptomatic patients. These findings support a selective, symptom-driven imaging strategy, although further prospective studies are needed to confirm safety and effectiveness.

## Simple Summary

The clinical benefit of routine chest X-rays performed immediately after minimally invasive pulmonary lobectomy, even in asymptomatic patients, is uncertain. We reviewed 554 patients who underwent video-assisted thoracoscopic lobectomy to assess whether routine X-rays in the recovery room changed management. Only 12 out of 554 (2.2%) led to any change in patient care; 1 patient required reoperation; 5 required chest tube reinsertions based on an X-ray finding without symptoms. Conversely, X-rays after chest tube removal more often led to clinical decisions. These findings support the consideration of a selective, symptom-driven imaging strategy in appropriately selected asymptomatic patients, although prospective validation is required.

## 1. Introduction

Postoperative chest radiography has long been a cornerstone of postoperative care in thoracic surgery. The traditional paradigm includes obtaining a chest X-ray (CXR) in the Post-Anesthesia Care Unit (PACU), followed by daily films during hospitalization and films before and after chest tube removal and even at outpatient follow-up [1,2,3]. This approach is historically tied to the imperative of rapidly identifying life-threatening complications such as pneumothorax, hemothorax, pleural effusions, or mispositioned drains in the immediate postoperative period [4]. However, despite continuous advancements in thoracic surgical techniques and perioperative management that have fundamentally modified patient risk profiles and recovery pathways, this protocol-driven approach persists. The constantly increasing volume of lung resections, combined with evolving perioperative care models, has led to reconsideration of the value of such systematic imaging in question [2,5,6].

The widespread adoption of video-assisted thoracoscopic surgery (VATS) and robotic-assisted thoracoscopic surgery (RATS) for pulmonary lobectomy represents a paradigm shift. Minimally invasive lobectomy, compared to open thoracotomy, is associated with significantly reduced surgical trauma, less postoperative pain, a lower incidence and duration of air leak, and faster functional recovery [7,8]. Concurrently, Enhanced Recovery After Surgery (ERAS) protocols have been implemented to standardize and optimize perioperative care, emphasizing evidence-based interventions and the systematic elimination of unnecessary procedures [5,6,9]. In this contemporary context, the patient undergoing VATS/RATS lobectomy is extubated in the operating room, often recovers in a standard ward without intermediate or intensive care, and is managed with a single, small-bore chest drain. These developments collectively challenge many of the traditional indications for routine postoperative imaging, such as the need to verify the position of multiple drains or central venous catheters, no longer utilized in standard VATS lobectomy [2,5,6].

### Study Aim and Hypothesis

The specific value of the first postoperative CXR in a homogeneous population of patients undergoing VATS lobectomy remains poorly defined. We hypothesize that in the context of elective, minimally invasive pulmonary lobectomy performed via VATS, the routine PACU CXR has an exceedingly low diagnostic and therapeutic yield. The primary aim of this retrospective cohort study is to quantify this yield, defined as the proportion of routine PACU CXRs that directly lead to a change in postoperative patient management (e.g., reintervention, chest tube reinsertion, computed tomography scan, unscheduled transfer in the ICU). Secondary aims include characterizing the spectrum of radiographic findings in the PACU setting and comparing the yield of routine PACU CXRs with that of routine post-drain-removal CXRs and evaluating the therapeutic yield of routine CXRs in patients with intraoperative complications. By providing procedure-specific data on a homogeneous cohort of VATS lobectomy patients, this study aims to inform evidence-based, value-driven imaging protocols aligned with ERAS principles, ultimately reducing unnecessary radiation exposure, costs, and workloads without compromising patient safety.

## 2. Materials and Methods

A retrospective, single-center, observational cohort study was conducted at a European tertiary referral center for thoracic surgery. The study was conducted in accordance with the Declaration of Helsinki. Due to the retrospective and non-interventional nature of the study, the requirement for ethical approval was waived, even though written informed consent was obtained from each patient. The manuscript was prepared following the Strengthening the Reporting of Observational Studies in Epidemiology (STROBE) guidelines, available in the Supplementary Materials.

### 2.1. Patient Population and Selection Criteria

All consecutive adult patients (≥18 years) who underwent elective pulmonary lobectomy via a VATS approach—either triportal, biportal, or uniportal—for primary lung cancer, pulmonary metastases, or benign conditions between 1 October 2014 and 31 December 2025 were identified from a prospectively maintained institutional surgical database. Exclusion criteria were conversion to thoracotomy, concomitant major procedures (e.g., chest wall resection, sleeve resection, diaphragmatic repair), emergency procedures, and missing or inaccessible postoperative imaging records or clinical data.

Our clinical institutional practice is to place one chest tube after lobectomy, through the utility incision in uniportal or biportal VATS and through the camera port in triportal VATS. All patients were transferred to the PACU immediately after surgery and underwent a routine postoperative CXR before being transferred to the ward on the same day. The chest tube was removed when no air leak was detected, and pleural effusion was less than 300 mL/24 h. After the first CXR in the PACU, patients underwent at least one CXR on the 3rd postoperative day (usually before chest tube removal) and after drainage removal (usually pre-discharge).

### 2.2. Data Collection

Data were manually extracted from electronic medical records using a standardized case report form and entered into a dedicated, password-protected database (Microsoft Excel, version 16.89.1 Microsoft Corporation, Redmond, WA, USA). Data on chest radiograph findings were collected by reviewing medical records and were based exclusively on the interpretations provided by two experienced thoracic radiologists (F.F. and P.F.). All CXRs were performed as routine examinations in asymptomatic patients, according to the institutional protocol. For the purpose of this study, “asymptomatic” was defined as the absence of documented clinical signs or symptoms at the time of CXR request, including dyspnea, hemodynamic instability, oxygen desaturation, an increased oxygen requirement, chest pain, or other specific clinical concerns noted by the attending surgical team. For every immediate postoperative and post-drainage removal CXR, we recorded the time point (PACU or post-drain removal) and radiographic findings (normal, pneumothorax with size, pleural effusion quantified based on the Blackmore score [10], atelectasis, subcutaneous emphysema, mispositioned drain, or other).

### 2.3. Outcomes and Definitions

The primary endpoint was the therapeutic yield of routine PACU CXRs, defined as the proportion of CXRs that directly led to a documented change in patient management. For the purpose of this study, a PACU CXR was defined as “expected postoperative” if it demonstrated only radiological signs typically observed after uncomplicated minimally invasive lobectomy (e.g., mild pleural effusion, small non-tension pneumothorax, minor atelectasis along the staple line, surgical emphysema, and proper chest tube and clip positioning), with no findings warranting active clinical intervention. Conversely, a CXR was considered “clinically actionable” if it revealed findings potentially requiring additional management, including large or tension pneumothorax, massive pleural effusion or hemothorax, contralateral pneumothorax, new extensive consolidation, mediastinal shift, or chest tube malposition requiring repositioning. A “change in management” was considered only if the CXR explicitly showed that the finding was the direct reason for initiating one of the following predefined actions: (I) surgical re-intervention; (II) chest tube insertion or re-insertion; (III) chest tube withdrawal or repositioning; (IV) computed tomography (CT) scan of the chest; (V) transfer to intensive care unit (ICU); (VI) repeat CXR (if explicitly justified by the index CXR finding); (VII) cancellation or delay of planned discharge (for CXR post-drain removal only).

The secondary outcomes were as follows: (I) therapeutic yield of post-drain-removal CXRs; (II) spectrum and frequency of radiographic findings in the PACU and post-drain-removal settings; (III) therapeutic yield of routine PACU CXRs associated with intraoperative complications.

The determination of whether a CXR was the direct reason for a management change was based on explicit documentation in the medical records by the attending surgeon. Two independent reviewers (F.F. and P.F.) reviewed the records to confirm the attribution. Any discrepancies were resolved based on a consensus.

### 2.4. Statistical Analysis

Categorical variables are presented as frequencies and percentages. Continuous variables are reported as the mean ± standard deviation (SD) if normally distributed or as the median and interquartile range (IQR) if non-normally distributed. Normality was assessed using the Shapiro–Wilk test. The primary endpoint was the therapeutic yield of routine PACU CXRs calculated as the proportion of examinations directly leading to a documented change in management. Exact 95% confidence intervals (CIs) for proportions were calculated using the Clopper–Pearson method. Comparisons of therapeutic yields between postoperative time points were performed using the McNemar test, as appropriate. The McNemar test was chosen because the same patients were evaluated at both time points (PACU and post-drain removal), making the observations paired. The test compares the proportion of patients with a management change at each time point while accounting for the paired nature of the data. For subgroup and temporal analyses, the Kruskal–Wallis and Mann–Whitney U tests were used as appropriate. A multiple linear regression model was fitted to identify independent predictors of the number of CXRs, including the year of surgery, duration of chest tube drainage, and length of hospital stay as covariates. Model assumptions were verified using the Shapiro–Wilk test and the Breusch–Pagan test. A *p*-value < 0.05 was considered statistically significant (two-tailed). No formal a priori sample size calculation was performed, as this was a retrospective analysis of a consecutive cohort. Statistical analysis was performed using StatPlus: MACPro Statistics, version 8.0 (2010 AnalystSoft Inc., Brandon, FL, USA).

## 3. Results

### 3.1. Patient Cohort and Baseline Characteristics

During the study period, a total of 554 consecutive patients underwent elective pulmonary lobectomy via a VATS approach (triportal, biportal, or uniportal) at our institution and met the inclusion criteria. The median age was 70 years (IQR: 63–76), and 315 (57%) were male. Surgical indication was primary non-small cell lung cancer (NSCLC) in 531 (96%), lung metastasis in 13 (2%), and benign disease in 10 (2%). A triportal VATS approach was used in 316 (57%) patients, while 154 (28%) underwent uniportal VATS and 84 (15%) biportal approach. Right-sided lobectomy was performed in 313 (56.5%). The median length of hospital stay was 6 days (IQR: 5–9; range 3–34 days). Baseline demographic and operative characteristics are summarized in Table 1.

### 3.2. Primary Outcome

#### Therapeutic Yield of Routine PACU CXRs

Of the 554 postoperative routine CXRs performed in the PACU, only 12 (2.2%; 95% CI: 1.1–3.7) led to a documented change in patient management according to our predefined criteria. The most common actions were chest tube reinsertion [n = 5 (42%)], followed by repeat CXR [n = 4 (33%)]. Chest tube withdrawal was performed in 2 patients and surgical re-intervention in 1 patient, with subsequent transfer to the ICU.

Of the 12 management changes triggered by routine PACU CXRs, 6 were classified as clinically meaningful interventions (chest tube reinsertion in 5 patients and surgical re-intervention in 1 patient). Overall, the management-change rate was 12/554 (2.2%), while the rate of clinically meaningful interventions was 6/554 (1.1%). The remaining 6 changes consisted of repeat CXR (n = 4) or chest tube withdrawal (n = 2), although repeat CXR may not represent a true therapeutic intervention.

All 5 chest tube reinsertions were performed for pneumothorax based solely on a routine PACU CXR finding in the absence of clinical symptoms (Table 2) with a median size of 3 cm (IQR: 2–5 cm). In 4 patients, the pneumothorax was apical, while in 1 patient it was localized laterally. None of these patients had evidence of tension physiology or hemodynamic compromise. All 5 patients underwent uneventful chest tube reinsertion and had an uncomplicated subsequent course. No patient required surgical re-intervention or ICU admission.

### 3.3. Secondary Outcomes

#### 3.3.1. Therapeutic Yield of Post-Drain Removal CXR

The therapeutic yield varied significantly across the two different postoperative time points: post-drain-removal CXRs, 93 out of 554 patients (16.8%; 95% CI: 13.7–20.0) required a change in management, compared to only 12 out of 554 (2.2%; 95% CI: 1.1–3.7) in the PACU setting (*p* < 0.001) (Table 3).

To further characterize the paired nature of these observations, we performed a McNemar test comparing management changes at the two time points within the same patients. The underlying 2 × 2 contingency table is presented in Table 4; the McNemar test was highly significant (*p* < 0.001), confirming that the difference between the two time points is statistically significant.

Among the 93 patients with a change in management after post-drain-removal CXRs, the most common actions were delayed discharge [n = 48 (51.6%)] and repeat CXR [n = 20 (21.5%)]. More invasive interventions, such as chest tube reinsertion [n = 4 (4.3%)] or surgical re-intervention [n = 1 (1.1%)], were rarely required. Details of the 93 cases with the therapeutic yield after chest-tube-removal CXRs are presented in Table 5.

Unlike the low-yield PACU CXRs, the higher diagnostic yield of post-drain-removal CXRs resulted in actionable clinical decisions, predominantly delayed discharge, allowing patients with radiographic abnormalities to be monitored or treated before discharge. None of the 93 patients required unplanned readmission.

#### 3.3.2. Spectrum and Frequency of Radiographic Findings in the PACU Setting Versus Post-Drain-Removal CXRs

Among the 554 routine PACU CXRs, 473 (85.4%) were reported as “expected postoperative” findings. The remaining 81 (14.6%) demonstrated at least one abnormal finding (some CXRs had multiple findings). Of these 81, 69 received no intervention whatsoever, and the finding was documented as “clinically insignificant” or “observation only.” The remaining 12 patients correspond to the cases with a change in management detailed in Table 3. The most frequent abnormal findings in PACU CXRs were pneumothorax [n = 32 (5.8%)], atelectasis [n = 25 (4.5%)], and pleural effusion [n = 11 (2.0%)].

By contrast, among the 554 routine post-drain-removal CXRs, 322 (58.1%) were reported as “expected postoperative,” while 232 (41.9%) demonstrated at least one abnormal finding. The most frequent abnormal findings were pleural effusion [n = 77 (33.2%)], opacities [n = 56 (24.1%), subcutaneous emphysema [n = 55 (23.7%)], and pneumothorax [n = 38 (16.4%)]. A comparative summary of radiographic findings between routine PACU CXRs and post-drain-removal CXRs is presented in Table 6. Statistical comparisons of individual radiographic findings were not performed due to the descriptive nature of this analysis and the potential for multiple findings in the same patient.

#### 3.3.3. Therapeutic Yield of Routine CXRs Associated with Intraoperative Complications

Intraoperative complications were recorded in 107 patients (19.3% of the cohort). These were classified as major (n = 27) and minor (n = 80). Major complications included bleeding >200 mL requiring intervention (n = 13), complete lymphadenectomy-related difficulties (n = 11), and bronchial injury (n = 3). Minor complications included air leaks (n = 68), treated with sealants, sutures, or fibrin glue, and other technical difficulties (n = 10), such as pericardial or diaphragmatic sutures (n = 2).

All 107 patients underwent a routine PACU CXR. Among these, only 4 patients (3.7%) had a change in management triggered by the PACU CXR, accounting for 4 of the 12 total management changes observed in the entire cohort (33.3%). Conversely, in the subgroup without intraoperative complications (n = 447), 8 patients (1.8%) had a change in management. Due to the small number of events in the complicated subgroup, a formal statistical comparison was not performed (Table 7).

Notably, even in patients with major intraoperative events, no surgical re-intervention was performed based solely on a routine PACU CXR finding in the absence of clinical symptoms. The four management changes observed in the complicated subgroup all consisted of chest tube reinsertion, with no further invasive procedures required. Due to the small number of events, these data are inconclusive and should be interpreted with caution. A formal statistical comparison was not performed.

### 3.4. Postoperative Chest X-Rays: Overview and Analysis

**Figure 1:**
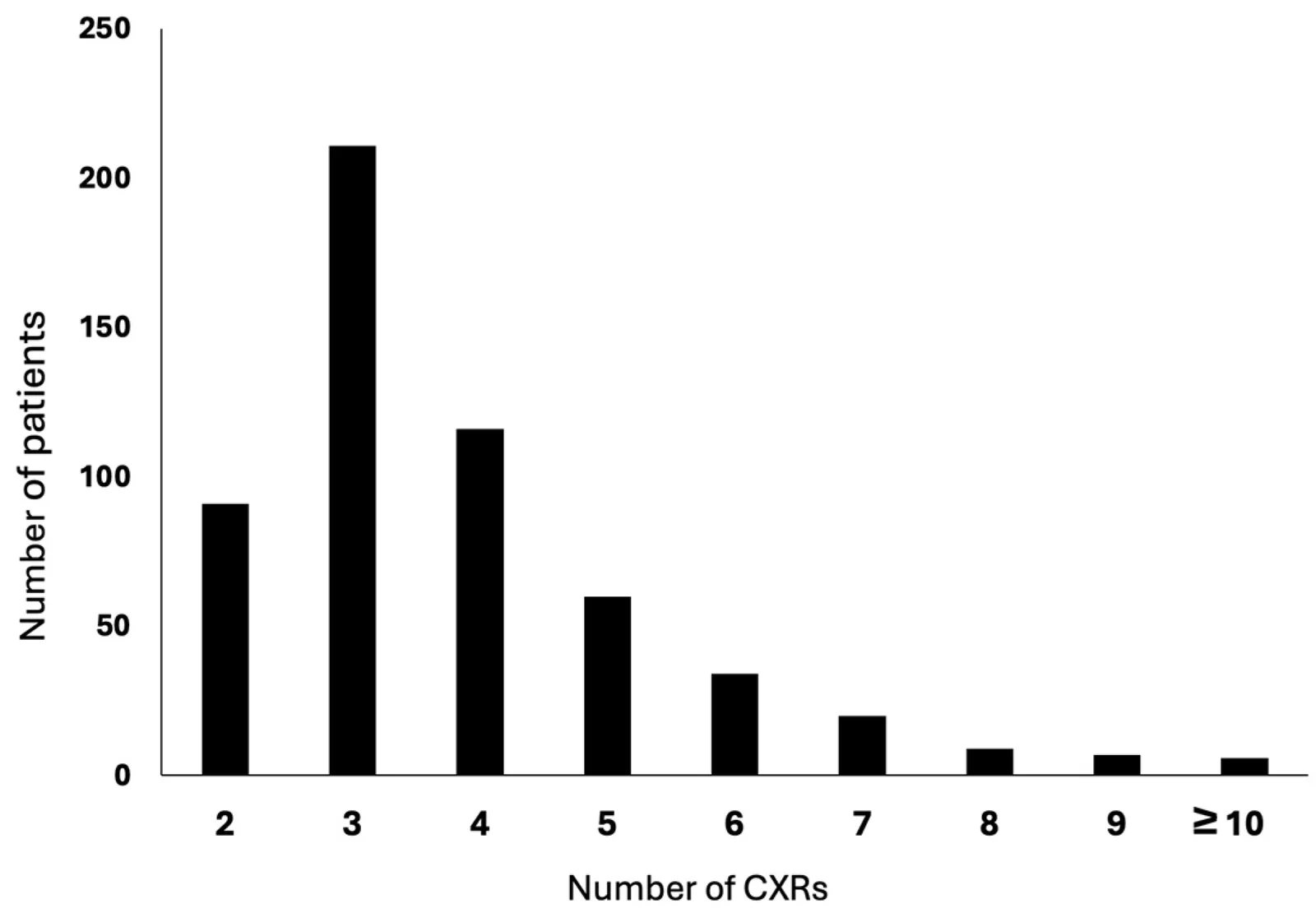
Distribution of the number of postoperative CXRs per patient (*N* = 554). The majority of patients (n = 210, 37.9%) underwent three CXRs. The distribution was right-skewed; six patients (1.1%) received 10 or more CXRs.

A total of 2130 postoperative CXRs were performed on the 554 patients during hospitalization, with a median of 3 CXRs per patient (IQR: 2–4; range: 2–18). The distribution was right-skewed, with a peak at 3 CXRs [(n = 210 (37.9%)] and the majority of patients [(n = 425 (76.7%)] receiving between 2 and 4 CXRs (Figure 1).

**Figure 2:**
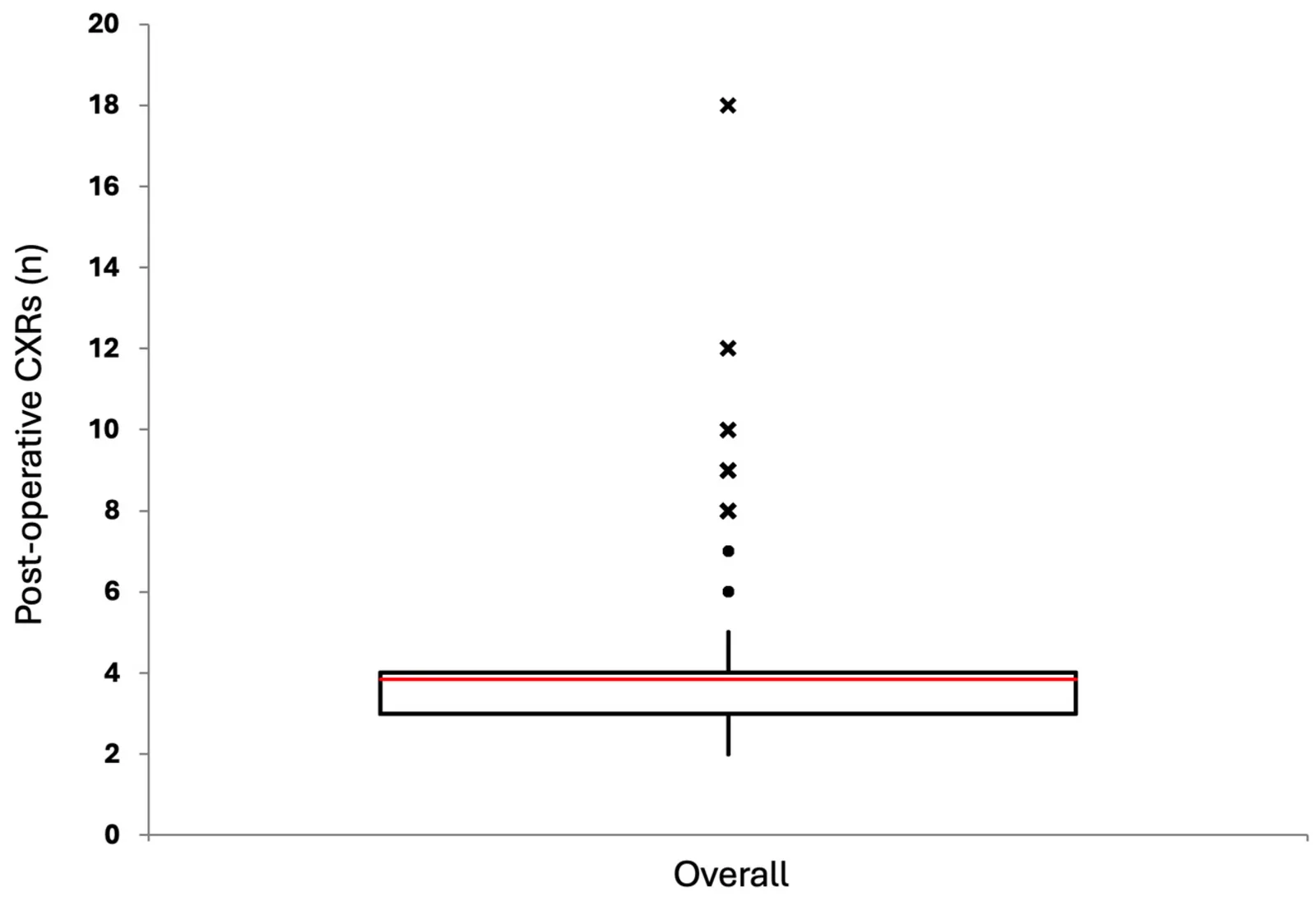
Box plot of the number of postoperative CXRs per patient (*N* = 554), showing the distribution of postoperative CXRs. The box spans the interquartile range (IQR) from Q1 = 3 to Q3 = 4, with the median at 3 (horizontal line). Whiskers extend to 1.5 × IQR from the hinges. Circles indicate outliers (>1.5 × IQR above the upper quartile), while crosses indicate extreme outliers (>3 × IQR above the upper quartile).

Box plot analysis identified six patients (1.1%) with 10 or more CXRs, representing the tail of the distribution and likely reflecting complicated postoperative courses (Figure 2).

**Figure 3:**
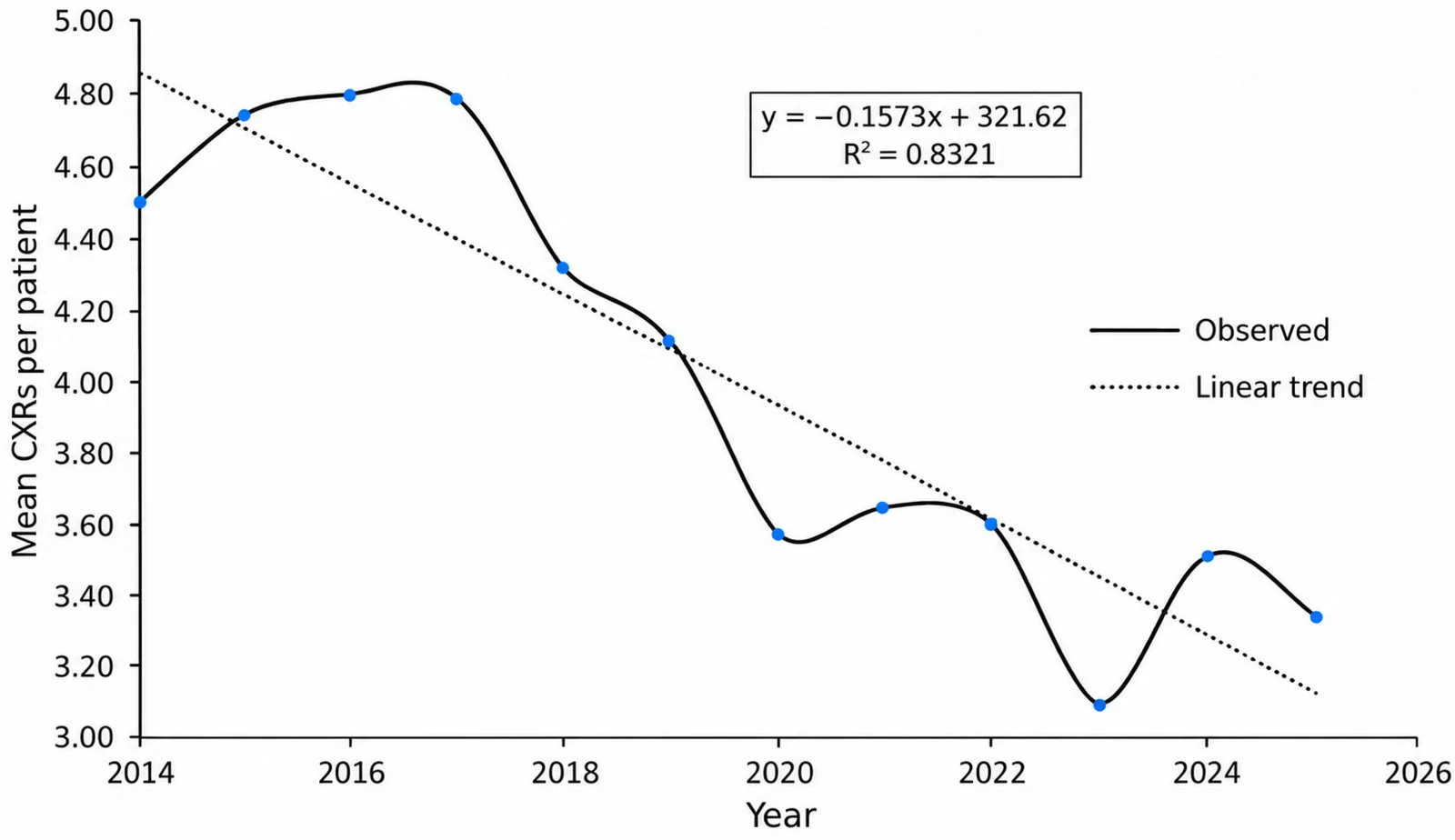
Temporal trend in mean postoperative CXRs per patient from 2014 to 2025. Line graph showing the annual mean number of postoperative CXRs per patient over the study period. The solid line connects observed annual means, while the dashed line represents the linear regression trend. A significant downward trend was observed (slope = −0.16 CXRs per year; 95% CI: −0.21 to −0.11; R^2^ = 0.83; *p* < 0.001).

Over the study period (2014–2025), a significant downward trend in CXR utilization was observed (Figure 3). The mean number of CXRs per patient decreased from 4.81 in 2016 to 3.31 in 2025 (slope = −0.16 CXRs per year; 95% CI: −0.21 to −0.11; R^2^ = 0.83; *p* < 0.001).

**Figure 4:**
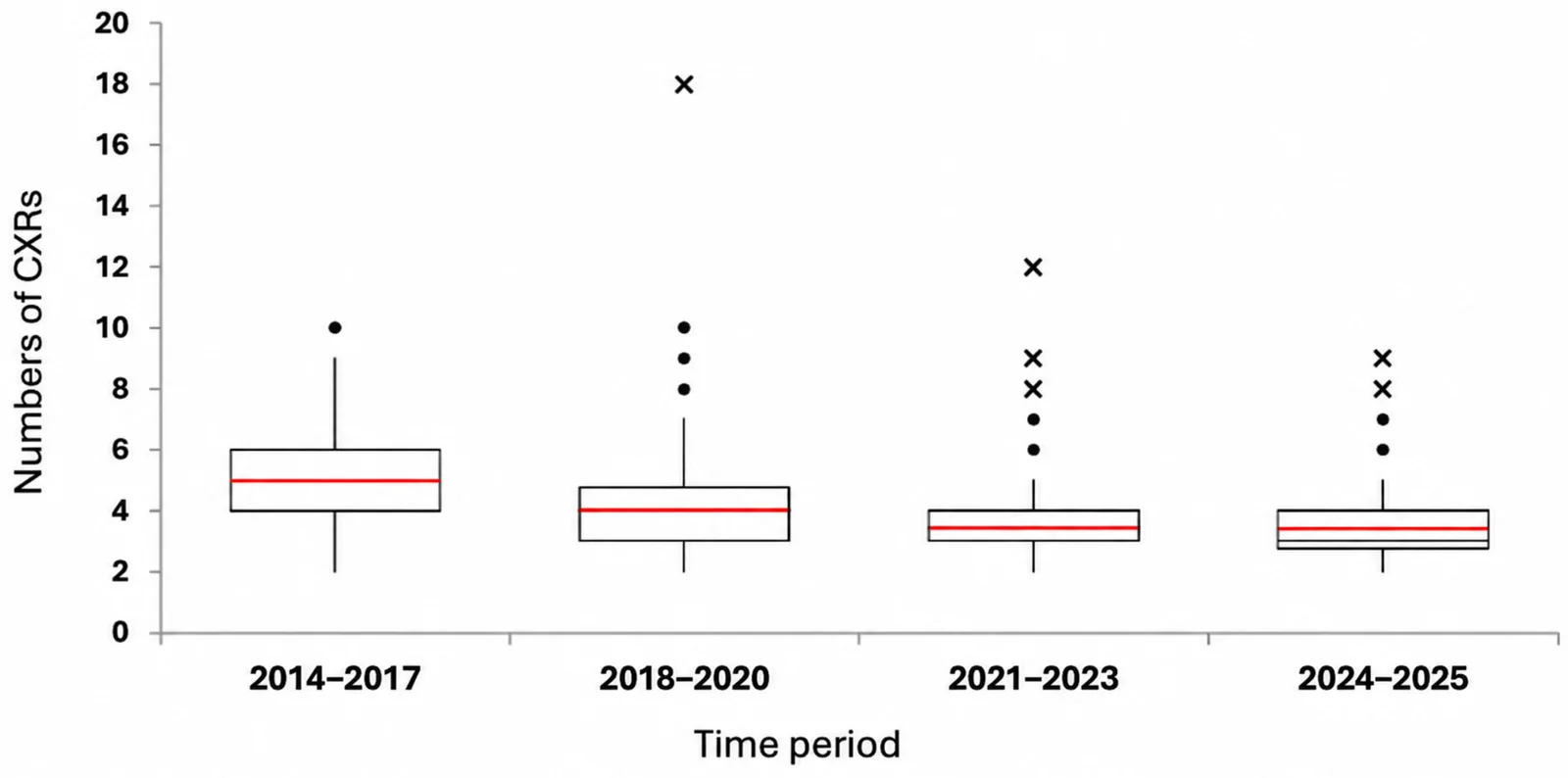
Box plots of postoperative CXRs per patient by period showing the distribution of postoperative CXRs per patient stratified by four time periods. Each box spans the interquartile range (IQR) with the median as a horizontal line; whiskers extend to the most extreme values within 1.5 × IQR. Circles represent outliers (>1.5 × IQR from the box), whereas crosses represent extreme outliers (>3 × IQR from the box). A progressive reduction in both the median (from 5.0 to 3.0) and the upper tail of the distribution is observed over time. However, outliers persist in all periods, indicating that a subset of patients with complicated postoperative courses continued to receive multiple CXRs.

When stratifying by period, the median number of CXRs decreased from 5 (IQR: 4–6) in 2014–2017 to 3 (IQR: 2.75–4) in 2024–2025 (Kruskal-Wallis H = 95.35; df = 3; *p* < 0.001) (Figure 4).

A multiple linear regression model confirmed that the year of surgery (β = −0.16; *p* < 0.001), duration of chest tube drainage (β = 0.12; *p* < 0.001), and length of hospital stay (β = 0.10; *p* < 0.001) were factors independently associated with the number of CXRs explaining 83% of the variance (R^2^ = 0.83) (Table 8).

### 3.5. Cost and Radiation Exposure Analysis

A hospital-perspective micro-costing analysis was performed using standard Italian public healthcare salary estimates for radiology technicians and radiologists, equipment amortization costs for digital radiography systems (assuming a 10-year lifecycle), maintenance costs, and PACS/RIS overhead. The estimated institutional cost per examination was as follows: €30 for portable PACU CXR (including personnel, mobile equipment amortization, maintenance, and PACS/RIS overhead); €20 for fixed radiology CXR performed after chest drain removal.

Radiation exposure was estimated using reference effective dose values for adult chest radiography: portable antero-posterior (AP) CXR, 0.05 mSv; standard AP and latero-lateral CXR, 0.08 mSv. All 554 patients underwent both examinations (total of 1108 radiographs), generating an estimated total institutional cost of €27,700 and an estimated cumulative radiation exposure of 72.02 mSv across the cohort. Per patient, the combined exposure was 0.13 mSv, corresponding to approximately 4–5% of the average annual natural background radiation in Europe. These estimates are based on institutional assumptions regarding personnel salaries, equipment amortization, and overhead and on reference effective doses rather than patient-specific dosimetry. They should therefore be considered approximate.

The cost per management change for PACU imaging, given a 2.2% intervention rate (12 cases out of 554 CXRs), was approximately €1385 per clinically relevant modification. When considering only clinically meaningful interventions (6 cases), the cost per clinically meaningful intervention was approximately €2770.

The cumulative cohort exposure corresponded to an estimated total dose of (0.07202 Sv) [11] (Table 9).

## 4. Discussion

This retrospective cohort study provides the first dedicated analysis of the isolated PACU CXR, focusing on a homogeneous cohort of patients undergoing VATS pulmonary lobectomy. Our findings demonstrate that routine CXR performed in the PACU has a very low therapeutic yield: only 2.2% of examinations directly led to a change in postoperative management, and these changes were predominantly minor, with few invasive procedures. This result aligns with and extends the growing evidence questioning routine immediate postoperative imaging in the era of minimally invasive thoracic surgery.

The immediate postoperative CXR is often justified as a routine safety check or “baseline”. However, its ability to detect clinically significant, unsuspected pathology that mandates immediate action is unclear. Previous studies on PACU CXRs are frequently pooled within larger cohorts that include wedge resections, segmentectomies, mediastinoscopies, and open approaches, making procedure-specific conclusions difficult [1,4]. Our study addresses this gap by isolating the PACU CXR in a homogeneous VATS lobectomy population.

The five asymptomatic patients who underwent chest tube reinsertion based on routine PACU CXR findings represent a clinically important subgroup. While these cases highlight the potential risk of omitting routine imaging, all patients were asymptomatic, and their management was driven solely by radiographic findings. The fact that none of these patients developed complications or required further interventions suggests that even in these cases, the clinical relevance of the detected pneumothoraces remains debatable.

Available evidence from mixed cohorts consistently shows low yields for routine CXRs. Bjerregaard et al. reported that only 0.9% of 1097 routine postoperative CXRs led to clinical intervention after elective VATS procedures [6]. Porter et al. found that PACU and post-drain-removal CXRs predominantly generated repeat imaging without translating into therapeutic interventions [3,12], while Whitehouse et al. confirmed that most abnormal routine CXRs did not alter management [13]. A meta-analysis by Galata et al. estimated a PACU CXR yield of 2.75% and a post-drain-removal yield of 7.3% across heterogeneous general thoracic surgery populations [1]. Our procedure-specific analysis shows a comparable lower PACU yield (2.2%), reinforcing the need for tailored rather than generic recommendations. The higher therapeutic yield of post-drain-removal CXRs may partly reflect differences in the outcome definition rather than a direct difference in diagnostic usefulness.

As shown in Figure 4, the median number of CXRs decreased from 5.0 in 2014–2017 to 3.0 in 2024–2025, consistent with the downward trend observed in Figure 3. Notably, the IQR also narrowed over time, from 4.0–6.0 to 2.5–4.0, suggesting a more standardized approach to postoperative imaging. However, outliers were present in all periods, with maximum values ranging from 9 to 18 CXRs, reinforcing the concept that a minority of patients with complex clinical courses still require intensive radiographic surveillance. The long study period (2014–2025) should be acknowledged as a potential source of confounding. During this time, significant changes occurred in VATS techniques, ERAS protocols, chest drain management, and institutional imaging practices. The observed decline in CXR utilization over time may therefore reflect these secular trends rather than a deliberate change in clinical practice. We also assessed whether the therapeutic yield of PACU CXR varied over time, but this analysis did not reveal significant changes.

Focusing on VATS lobectomy, our results are consistent with those of Hsu et al., who observed that asymptomatic patients with abnormal post-drain-removal CXRs required no intervention, whereas symptomatic patients often needed procedures regardless of the radiographic findings [7]. Even more compelling, Alcasid and coworkers demonstrated that postoperative signs and symptoms predicted the need for intervention with an odds ratio of approximately 48, while an abnormal postoperative day-1 CXR was not an independent predictor [2]. These observations are supported by Whitehouse et al., who emphasized that clinically relevant events are almost always preceded by symptoms rather than isolated radiographic abnormalities [13].

Quality improvement initiatives have further shown that eliminating routine CXRs after lung resection is safe, significantly reducing the number of examinations without increasing complications or readmissions [14]. Table 10 summarizes key literature on routine postoperative CXRs in thoracic surgery.

Meanwhile, Table 11 provides a direct comparison of studies specifically addressing PACU CXRs.

Previous investigations have examined later time points such as post-drain removal [7] or post-operative day 1 (POD1) [2], and meta-analyses have pooled heterogeneous populations [1]. In contrast, our study isolates and quantifies the therapeutic yield of the immediate PACU CXR in a homogeneous VATS lobectomy cohort. Our observed yield of 2.2% is consistent with the pooled estimate of 2.75% from Galata et al. and with the 0.4–0.9% range reported in mixed cohorts [1]. This consistency across different settings reinforces the robustness of the evidence and supports the feasibility of a selective, symptom-driven imaging strategy in asymptomatic patients. Our secondary analysis of post-drain-removal CXRs aligns with the extensive literature showing that post-pull pneumothorax is common but almost always clinically irrelevant. Dezube et al. found that 55% of patients after minimally invasive resections had a small pneumothorax, with none requiring intervention [8]. Zukowski et al. reported that 33.2% of post-pull CXRs were abnormal, but only 3% required drain reinsertion, and all were symptomatic [9]. Some authors confirmed that a symptom-only strategy does not increase complications or readmissions [17,18]. These findings collectively argue that routine post-drain-removal CXRs in asymptomatic VATS patients are low-value and should be reserved for symptomatic patients or those with high-risk features (e.g., prolonged air leak, positive clamp trial).

Although our study did not specifically address daily CXRs, the literature consistently indicates that routine daily CXRs after pulmonary resection have limited value in non-hypoxic patients and can be safely replaced by an on-demand strategy [1,13]. Our finding that even the first postoperative CXR has a low yield indirectly supports the view that subsequent daily CXRs are even less justifiable, consistent with the literature.

### 4.1. Safety, Cost, and Radiation Considerations

One of the most consistent messages from the literature is that eliminating routine CXRs is safe, since reducing CXR utilization does not increase complications or readmissions [3,14,17]; Porter et al. reported reductions of >25–40% in CXR use [12]. Our economic analysis (Section 3.5) estimated a total institutional cost of €27,700 for routine PACU and post-drain-removal CXRs, with an approximate cost per PACU management change of €1385. Combined with an estimated cumulative radiation exposure of 72.02 mSv (0.13 mSv per patient) and a near-zero clinical yield, these findings do not support the routine use of PACU imaging in asymptomatic patients. They align with radiation management principles and the growing emphasis on value-based perioperative care and are consistent with the growing body of literature demonstrating that eliminating routine imaging does not increase complications or readmissions [3,12,14,17]. However, these estimates are based on institutional assumptions and the reference dose value and should therefore be interpretated as approximate rather than patient-specific measurements.

An emerging alternative is chest ultrasound (CUS). Grapatsas et al. documented that selective CUS can reduce postoperative CXRs by up to 60%, with therapeutic concordance rates as high as 97%, reserving CXR for cases of massive subcutaneous emphysema, complex effusions, or inconclusive ultrasound findings [15].

### 4.2. Study Limitations

Our study has several limitations. First, as a retrospective single-center design, inherent biases and dependence on accurate documentation cannot be eliminated, and results may not be generalizable to centers with different protocols or case mixes. Second, no formal sample size calculation was performed. However, the sample size provided a reasonably precise estimate of the primary endpoint, as reflected by the 95% confidence interval (2.2%, 95% CI: 1.1–3.7). Nevertheless, it was insufficient to robustly analyze subgroups. Third, our analysis was limited to routine CXRs performed in asymptomatic patients; we did not evaluate the yield of CXRs obtained for clinical indications, which may have a different profile. Additionally, we did not systematically collect data on chest ultrasounds or patient-reported outcomes, which represent important directions for future research.

Prospective, randomized trials focused on specific procedures (VATS lobectomy) and specific time points (PACU, post-drain removal, follow-up) are needed to consolidate recommendations into formal guidelines. Nevertheless, this is one of the few studies focusing exclusively on VATS lobectomy, providing procedure-specific rather than pooled data and using a predefined definition of “therapeutic yield” to avoid overestimating the clinical impact. Our cohort reflects real-world contemporary practice in a high-volume thoracic surgery center. In conjunction with the extensive literature reviewed, our findings support a paradigm shift in postoperative imaging after VATS lobectomy.

## 5. Conclusions

Routine CXRs in the PACU following VATS lobectomy have minimal therapeutic yields and rarely alter clinical management in asymptomatic patients. The literature consistently demonstrates that clinical signs and symptoms are far more predictive of the need for intervention than routine radiographic findings. Our findings, in conjunction with the available literature, support the evaluation of a more selective, symptom-driven imaging strategy. Prospective studies are needed to confirm the safety and effectiveness of this approach in appropriately selected patients.

## Figures and Tables

**Table 1:** Baseline characteristics of the study population (*N* = 554).

Characteristic	Value
Age, years, median (IQR)	70 (63–76)
Sex, male, n (%)	315 (57)
Side of resection	n (%)
*Right*	313 (56.5)
Surgical indication	n (%)
*NSCLC*	531 (96)
*Metastasis*	13 (2)
*Benign disease*	10 (2)
VATS approach	n (%)
*Triportal*	316 (57)
*Uniportal*	154 (28)
*Biportal*	84 (15)
Site of surgery	n (%)
*Right upper lobe*	165 (30)
*Middle lobe*	39 (7)
*Right lower lobe*	102 (18)
*Bilobectomy*	8 (1)
*Left upper lobe*	144 (26)
*Left lower lobe*	96 (17)
Intraoperative complications (Minor and major)	107 (19.3)
Length of stay, days, median (IQR), range	6 (5–9); 3–34 days
Postoperative CXRs (554 pts), n. IQR range	2130 (3–4) 2–18

IQR: interquartile; NSCLC: non-small cell lung cancer; VATS: video-assisted thoracic surgery; CXRs: chest X-rays.

**Table 2:** Changes in management triggered by 12 of 554 routine PACU *CXRs*.

Action Taken	*N* (%)
Chest tube reinsertion	5 (42)
Repeat CXR *	4 (33)
Chest tube withdrawal	2 (17)
Surgical re-intervention	1 (8)
Total actions	12 (100)

* Repeat CXR is not clinically meaningful intervention.

**Table 3:** Therapeutic yield of routine CXRs by time point: PACU vs. post-drain removal.

Time-Point	CXRs n	CXRs with Change of Management n (%)	95% CI	*p*-Value
PACU (routine)	554	12 (2.2)	1.1–3.7	<0.001
Post-drain removal	554	93 (16.8)	13.7–20.0	

CXRs: chest X-rays; CI: confidence interval; PACU: Post-Anesthesia Care Unit.

**Table 4:** Paired comparison of management changes between PACU and post-drain removal of 554 CXRs.

	Post-Drain Removal: Change	Post-Drain Removal: No Change	Total
PACU: Change	4	8	12
PACU: No Change	89	453	542
Total	93	461	554

McNemar test: *p* < 0.001.

**Table 5:** Changes in management triggered by 93 of 554 routine post-drain-removal CXRs.

Action Taken	*N* (%)
Delayed discharge	48 (51.6)
Repeat CXR	20 (21.5)
Chest tube withdrawal or Heimlich valve placement	5 (5.4)
Chest tube reinsertion	4 (4.3)
CT scan	2 (2.2)
Surgical re-intervention	1 (1.1)
Other *	13 (14)
Total	93 (100)

Note: some CXRs had multiple findings. * Other actions included the following: drain removal (n = 4), abdominal X-ray (n = 1), and other non-specified conservative measures (n = 8).

**Table 6:** Comparison of radiographic findings between routine PACU CXRs and post-drain-removal CXRs.

Finding	PACU CXRs n. (%)	Post-Drain-Removal CXRs n. (%)
Expected post-operative CXRs	473 (85.4)	322 (58.1)
Abnormal findings *	81 (14.6)	232 (41.9)
Pneumothorax ^†^	32 (5.8)	38 (6.8)
*Small (≤2 cm apical)*	*20 (62.5)*	*23 (60.5)*
*Moderate (>2 cm)*	*10 (31.3)*	*9 (23.7)*
*Large (≥50% hemithorax)*	*2 (6.3)*	*6 (15.8)*
Pleural effusion **	11 (1.9)	77 (33.2)
Atelectasis	25 (4.5)	56 (24.1)
Subcutaneous emphysema	6 (1.1)	55 (23.7)
Mispositioned drain	3 (0.5)	- ^††^
Elevated hemidiaphragm	4 (0.7)	6 (1.1)

Note: some CXRs had multiple findings. * Percentages calculated based on total 554 CXRs; ^†^ percentage calculated based on total of pneumothoraces; ** pleural effusion was quantified based on the Blackmore score; ^††^ not observed in this setting.

**Table 7:** Therapeutic yield of routine PACU CXRs by intraoperative complication status.

Intraoperative Complications	Patients, n	Routine PACU CXRs, n	CXRs with Change, n (%)
Yes	107	107	4 (3.7)
No	447	447	8 (1.8)
Total	554	554	12 (2.2)

**Table 8:** Multiple linear regression analysis of factors associated with the number of postoperative CXRs.

Variable	Coefficient (β)	95% CI	*p*-Value
Year of surgery	−0.16	−0.21 to −0.11	<0.001
Duration of chest tube drainage	+0.12	+0.08 to +0.16	<0.001
Length of hospital stay	+0.10	+0.06 to +0.14	<0.001

Note: R^2^ = 0.83; Adjusted R^2^ = 0.82.

**Table 9:** Combined economic and radiation burden.

Examination Type	No. of Exams	Cost per Exam (€)	Total Cost (€)	Dose per Exam (mSv)	Total Dose (mSv)	Management Change Rate (%)
Portable CXR (PACU)	554	30	16,620	0.05	27.7	2.2
Fixed CXR (post-drain)	554	20	11,080	0.08	44.32	16.8
Total	1.108	50	27,700	—	72.02	—

**Table 10:** Key evidence from the recent literature on routine postoperative CXRs in thoracic surgery.

Focus	Main Message	Key Studies
Post-pull CXR after minimally invasive resection	Pneumothoraces are frequent but never require intervention in asymptomatic patients	Dezube, 2021 [8]; Hsu, 2022 [7]; Zukowski, 2022 [9]
Routine CXR after elective VATS	Only 0.9% lead to intervention; routine abolition suggested	Bjerregaard, 2015 [6]
Perioperative CXR in thoracic surgery (meta-analysis)	No evidence for preoperative or daily CXRs; selective utility only	Galata, 2022 [1]
Routine PACU and post-drain-removal CXR	Frequent abnormalities, no procedures; minimal clinical impact	Porter, 2020 [3]
Quality improvement/implementation	Eliminating routine CXRs does not increase complications or readmissions	Vasey, 2025 [14]
Chest ultrasound (CUS)	Can reduce CXRs by up to 60% with high therapeutic concordance	Grapatsas, 2022 [15]

**Table 11:** Summary of available evidence on routine PACU CXRs after thoracic surgery, with a specific focus on VATS lobectomy.

Study	Study Design	Population	n pts (n CXR)	Therapeutic Yield PACU CXR (%)
Bjerregaard 2015 [6]	Retrospective observational	Mixed elective VATS surgery (all procedures)	1002 (1097)	0.9%
Porter 2020 [3]	Retrospective	General thoracic surgery mixed procedures (VATS/Open)	241 (482)	0.4%
Galata 2022 [1]	Review and meta-analysis	General thoracic surgery mixed procedures	3841 (4784)	2.75% (pooled PACU estimate)
Alcasid 2024 [2]	Retrospective	VATS/RATS lobectomies	362 (NA) ^§^	NA for PACU ^0^
HSU 2022 [7]	Retrospective	VATS lobectomies	208 (NA) ^§^	NA for PACU ^0^
Cerfolio 2011 [16]	Retrospective	Pulmonary thoracotomic resection	1037 (1037)	NA
Rotolo 2026 *	Retrospective	VATS lobectomies	554 (554)	2.2% (95% CI: 1.1–3.7)

NA: not available/not reported separately for PACU time point; ^§^ all postoperative CXRs; ^0^ PACU not isolated. * Present study.

## Data Availability

The anonymized datasets generated and analyzed during the current study are available from the corresponding author upon reasonable request, due to ethical restrictions related to patient privacy and in accordance with national data protection laws.

## Supplementary Materials

The following supporting information can be downloaded at: https://www.mdpi.com/article/10.3390/tomography12090136/s1, STROBE Statement.

## Abbreviations

The following abbreviations are used in this manuscript:

VATS	Video-assisted thoracic surgery
ERAS	Enhanced Recovery After Surgery
CXRs	Chest X-rays
PACU	Post-Anesthesia Care Unit
CI	Confidence interval
RATS	Robotic-assisted thoracic surgery
ICU	Intensive care unit
CT	Computed tomography
SD	Standard deviation
IQR	Interquartile
NSCLC	Non-small cell lung cancer
R^2^	Coefficient of determinations
€	Euro
mSv	milliSievert
