# Supplementary material for: Routine Immediate Postoperative Chest Radiography After Minimally Invasive Pulmonary Lobectomy Has a Low Impact on Clinical Management: A Value-Based Imaging Analysis
Source: Tomography. 2026 Sep 17;12(9):136. doi: 10.3390/tomography12090136 (PMC13611314; doi:10.3390/tomography12090136)
Supplement: Supplementary file 1 [file tomography-12-00136-s001.zip › tomography-4495259-supplementary.pdf]

STROBE Statement: Checklist of items that should be included in reports of cohort studies

	Item No	Recommendation	Section/Page	Notes
Title and abstract	1	(a) Indicate the study's design with a commonly used term in the title or the abstract	Abstract	"retrospective, single-center study"
		(b) Provide in the abstract an informative and balanced summary of what was done and what was found	Abstract	Background, Methods, Results, Conclusions
Introduction				
Background/rationale	2	Explain the scientific background and rationale for the investigation being reported	Introduction	See Introduction section (paragraphs 1-3) Traditional practice, VATS/ERAS evolution, evidence against routine CXRs
Objective	3	State specific objectives, including any prespecified hypotheses	Introduction (1.2)	"We hypothesize that... The primary aim is to quantify this yield..."
**Methods**				
Study design	4	Present key elements of study design early in the paper	Methods section 2	"single-center, retrospective, observational cohort study"
Setting	5	Describe the setting, locations, and relevant dates, including periods of recruitment, exposure, follow-up, and data collection	See Methods section	"Tertiary referral center", "January 2014 – December 2025", "consecutive patients"
Participants	6	(a) Give the eligibility criteria, and the sources and methods of selection of participants	Methods	Inclusion: ≥18 yrs, elective VATS lobectomy. Exclusion criteria listed.
		(b) For matched studies, give matching criteria and number of exposed and unexposed	Not Applicable	No matched study
Variables	7	Clearly define all outcomes, exposures, predictors, potential confounders, and effect modifiers. Give diagnostic criteria, if applicable	See Methods section and Table 2	"Therapeutic yield" defined with 6 predefined actions. Time-points, indications, findings

	Item No	Recommendation	Section/Page	Notes
Data sources/measurement	8	For each variable of interest, give sources of data and details of methods of assessment (measurement). Describe comparability of assessment methods if there is more than one group	Methods section	"Data extracted from electronic medical records using standardized case report form"
Bias	9	Describe any efforts to address potential sources of bias	See Methods section	Stringent exclusion criteria. Rigorous definition of "therapeutic yield" to avoid overestimation
Study size	10	Explain how the study size was arrived at	Methods (2.4)	"No formal a priori sample size calculation... consecutive cohort"
Quantitative variables	11	Explain how quantitative variables were handled in the analyses. If applicable, describe which groupings were chosen and why	Se Methods section 2, subsection 2.4	Categorical: frequencies/percentages. Continuous: mean±SD or median (IQR)
Statistical methods	12	(a) Describe all statistical methods, including those used to control for confounding	Se Methods section 2, subsection 2.4	Chi-square/Fisher, Clopper-Pearson per CI 95%
		(b) Describe any methods used to examine subgroups and interactions	Methods 2.4)	Comparisons by time-point (PACU vs. post-drain removal). Two subgroup
		(c) Explain how missing data were addressed	See methods section	Patients with missing data were excluded
		(d) If applicable, explain how loss to follow-up was addressed	Not Applicable	Retrospective study
		(e) Describe any sensitivity analyses	Section 3.5.	Cost analysis with sensitivity ranges
**Results**				
Partecipants	13	(a) Report numbers of individuals at each stage of study—eg numbers potentially eligible, examined for eligibility, confirmed eligible, included in the study, completing follow-up, and analysed	See Results section (3.1)	553 patients included. Exclusion criteria mentioned
		(b) Give reasons for non-	Results (3.1)	"After applying exclusion

	Item No	Recommendation	Section/Page	Notes
		participation at each stage		criteria..."
		(c) Consider use of a flow diagram	N/A	Not added
Descriptive data	14	(a) Give characteristics of study participants (eg demographic, clinical, social) and information on exposures and potential confounders	See Results section and Table 1	Age, sex, side, indication, VATS approach, LOS
		(b) Indicate number of participants with missing data for each variable of interest	Results 3.1)	Missing data = excluded patients
		(c) Summarise follow-up time (eg, average and total amount)	Results (3.1)	Median length of stay: 6 days IQR: 5-9.
Outcome data	15	Report numbers of outcome events or summary measures over time	See Results sections and Table 2	12 out of 554 PACU CXRs led to management change
Main Results	16	(a) Give unadjusted estimates and, if applicable, confounder-adjusted estimates and their precision (eg, 95% confidence interval). Make clear which confounders were adjusted for and why they were included	Results (3.2, 3.3.1 and Table 7)	Yield, Cis and regression coefficient.
		(b) Report category boundaries when continuous variables were categorized	Not Applicable	Not Applicable
		(c) If relevant, consider translating estimates of relative risk into absolute risk for a meaningful time period	Not Applicable	Not Applicable
Other analyses	17	Report other analyses done—eg analyses of subgroups and interactions, and sensitivity analyses	See Results section 3.3.3, 3.4 and 3.5)	Intraoperative complications, spectrum of findings, cost analysis, temporal trend
**Discussion**				

	Item No	Recommendation	Section/Page	Notes
Key results	18	Summarise key results with reference to study objectives	See Discussion section (first paragraph)	Very low yield, minor changes, never invasive procedures
Limitations	19	Discuss limitations of the study, taking into account sources of potential bias or imprecision. Discuss both direction and magnitude of any potential bias	See Discussion section sbsection 4.3	Retrospective, single-center, subgroup sample size, lack of standardization for "clinically indicated" CXRs
Interpretation	20	Give a cautious overall interpretation of results considering objectives, limitations, multiplicity of analyses, results from similar studies, and other relevant evidence	Discussion	Comparison with Bjerregaard, Porter, Galata, Hsu, Alcasid et other authors. Confirmation of existing literature
Generalisabilty	21	Discuss the generalisability (external validity) of the study results	See Discussion 4.2	"May not be generalizable…"
**Other information**				
Funding	22	Give the source of funding and the role of the funders for the present study and, if applicable, for the original study on which the present article is based	Funding section	"This research received no external funding"
